# Institutional Incidence of Severe tPA-Induced Angioedema in Ischemic Cerebral Vascular Accidents

**DOI:** 10.1155/2018/9360918

**Published:** 2018-09-27

**Authors:** Matthew Sczepanski, Paul Bozyk

**Affiliations:** Beaumont Health–Royal Oak, 3601 W. 13 Mile Rd., Royal Oak, MI 48073, USA

## Abstract

**Introduction:**

Tissue plasminogen activator (tPA) is commonly used in ischemic cerebral vascular accidents (CVAs). tPA is generally well tolerated; however, orolingual angioedema is a well-documented adverse effect. Angioedema is generally mild, transient, and unilateral but can manifest as severe, life-threatening upper airway obstruction requiring intubation. Reported incidence for all severities ranges from one to five percent, whereas reported incidence of severe cases ranges from 0.18 to 1 percent of patients receiving tPA for ischemic CVA. Angiotensin-converting enzyme (ACE) inhibitors and middle cerebral artery distribution have been associated with a higher risk of developing angioedema. The aim of this study is to evaluate the incidence of severe tPA-induced angioedema and its effects on length of stay (LOS) and death.

**Methods:**

A retrospective chart review of patients receiving tPA for ischemic CVA from January 2014 through December 2016 was conducted at a large tertiary center with Comprehensive Stroke Center designation. Subjects were eighteen or older. Baseline demographics and clinical data were collected.

**Results:**

147 patients were included with four developing severe angioedema due to tPA resulting in an incidence of 2.72%. All four were female. The median LOS was thirty days for patients with angioedema and twelve days for those without. The survival probability was higher in the angioedema group and mean time to death was twenty-two days in the angioedema group and twenty-one days in the nonangioedema group. Twenty-five patients died, one from the angioedema group. ACE inhibitor use was found to have an OR of 7.72.

**Conclusion:**

This study found a higher incidence of severe angioedema than that reported. Development of severe angioedema increased length of stay but was not shown to worsen outcomes in regards to death. Consistent with previous studies, ACE inhibitor use was associated with a higher risk of developing angioedema.

## 1. Introduction

Tissue plasminogen activator (tPA) is commonly used in patients that present with acute ischemic cerebral vascular accidents (CVAs). tPA is generally well tolerated and can be life-saving in carefully selected patients. The main concerning adverse effect of tPA is cerebral hemorrhage; however, another well-known side effect is orolingual angioedema. The pathophysiology of tPA-induced angioedema is not fully understood but is thought to be due to activation of both the kinin pathway as well as the complement pathway.

Hydrolysis of plasminogen to plasmin by tPA activates the kinin pathway by converting Factor XII to Factor XIIa which will cause an increase in production of bradykinin. Bradykinin is a powerful vasodilator peptide that activates vascular bradykinin B2 receptors which are responsible for causing angioedema. Bradykinin B2 receptors are upregulated in dying neurons such as that seen in ischemic CVA [1]. Angiotensin-converting enzyme (ACE) inhibitors prevent bradykinin degradation allowing for accumulation of bradykinin that can cause angioedema, and it is well known that ACE inhibitor use is associated with a higher risk of developing angioedema with tPA administration [1–6].

Moreover, it is proposed that tPA activates the complement pathway by increasing C4a and C5a. Increased levels of C4a and C5a increase mast cell and basophil degranulation of vasoactive substances that contribute to angioedema [7]. Nevertheless, the kinin pathway is thought to play a larger role in tPA-induced angioedema than the complement pathway.

Symptoms of tPA-induced angioedema are typically mild, transient, unilateral swelling of the tongue and lips [1]. However, in some cases, angioedema can progress to severe, life-threatening upper airway obstruction that may necessitate intubation. The majority of reports place the overall incidence between one to five percent for all patients receiving tPA for CVA indications [2–15] with one study having a reported incidence as high as 7.9 percent [1] and four studies with an incidence less than one percent [16–19]. This overall incidence encompasses all severities. To date, severe cases are believed to be rare with an incidence ranging from 0.18 to one percent of all patients receiving tPA, as noted in the applicable studies [1–5, 10, 13, 17]. The remaining studies available on this topic fail to characterize the severity of angioedema which could result in an inaccurate incidence of severe tPA-induced angioedema [6–9, 11, 12, 14–16, 18, 19]. Furthermore, there are no studies to date that evaluate the effects of length of stay and death in patients that develop severe tPA-induced angioedema. This study aims to fill these gaps by evaluating the incidence of severe tPA-induced angioedema in a large academic tertiary care hospital compared to that found in the literature in addition to evaluating its effects on length of stay and death.

## 2. Methods

A retrospective chart review was performed for all patients that were treated with intravenous (IV) tPA for ischemic CVA at Beaumont Health–Royal Oak in Royal Oak, MI, from January 2014 through December 2016. Beaumont Health–Royal Oak is a 1,070 bed tertiary care center and in 2013 was designated a Comprehensive Stroke Center by The Joint Commission.

To be eligible, patients needed to be age eighteen or older and been treated with IV tPA for a suspected diagnosis of acute ischemic CVA on presentation or during their hospital admission. Patients were deemed eligible for tPA based on the American Stroke Association eligibility criteria and symptom onset within four and a half hours from tPA administration. Administration of tPA for CVA indication was as an initial bolus of 0.09 mg/kg followed by an infusion of 0.81 mg/kg over one hour, per hospital protocol. All patients were monitored for at least twenty-four hours in the intensive care unit following tPA administration. The patients were identified by electronic medical record (EPIC) for tPA administration for CVA indication. Baseline demographics were collected that included age, sex, race, cardiovascular risk factors, and ACE inhibitor use. Clinical data were collected that included the area of infarct based on CT or MRI, need for intubation if development of angioedema, baseline National Institutes of Health Stroke Scale (NIHSS) score, time from onset of symptoms to administration of tPA, length of stay (hospital, hospice, and total which is defined as the sum of hospital and hospice length of stay), documentation of a C1-esterase deficiency status, and death during admission. As there is no current hospital protocol in place requiring documentation of angioedema development at scheduled intervals and based on the study design of a retrospective chart review, milder cases would likely be omitted and only severe angioedema cases would be identified. Severe angioedema was defined as angioedema causing life-threatening airway obstruction with or without the need for intubation. Patients developing angioedema within twenty-four hours after administration of tPA were identified as per Huford et al. [1].

## 3. Statistical Analyses

Categorical variables were summarized with counts and percentages. Nonnormally distributed continuous variables were summarized with medians and ranges. Normally distributed variables were summarized with mean and standard deviation. A 95 percent confidence interval for the primary outcome of severe angioedema was obtained using the Wilson (score) method. Kaplan–Meier survival curves were obtained for mortality; patients who were discharged alive and not sent to hospice were treated as censored at the length of hospital stay for this analysis. For the patients that developed severe angioedema, odds ratios for severe angioedema development were calculated for ACE inhibitor use and comorbidities. Due to the small number of patients developing severe angioedema, p values were not calculated for the comparison of patients with and without severe angioedema on various factors. The SAS system for Windows version 9.3 was used for all statistical analysis.

## 4. Results

One hundred forty-nine encounters were identified during this time period that met the inclusion criteria. Two patients were found to have two separate encounters that fit inclusion; only their initial encounter was used so that the assumption of statistical independence was reasonable. As a result, one hundred forty-seven encounters were included in the analyses.  Table 1 shows the baseline characteristics by angioedema development status. There were no known patients with documented C1-esterase deficiency.

Five patients had documented severe angioedema during their admission. One patient developed severe angioedema five days after administration of tPA; for this patient, angioedema was thought to be due to an antibiotic and was not included in the calculation of incidence of severe tPA-induced angioedema. Therefore, four patients developed severe angioedema within twenty-four hours of tPA administration and all four cases required intubation. This resulted in an incidence of 2.7% (95% CI: 1.1% to 6.8%) for severe tPA-induced angioedema. All four patients were female. Odds ratios for ACE inhibitor use and comorbidities for patients that developed severe angioedema are displayed in Table 2. Three of the patients were on ACE inhibitors with an odds ratio of 7.73. Three of the four patients had areas of ischemia in the middle cerebral artery territory. Additionally, one patient that developed severe angioedema died during their hospital admission.

Compared to patients who did not develop angioedema, the severe angioedema group's mean age was about ten years older than those without angioedema. Mean time from onset of CVA symptoms to tPA administration was less in the severe angioedema group (118.3 min. vs 140.9 min). In both groups, the majority of CVAs were in the middle cerebral artery distribution.

**Figure 1:**
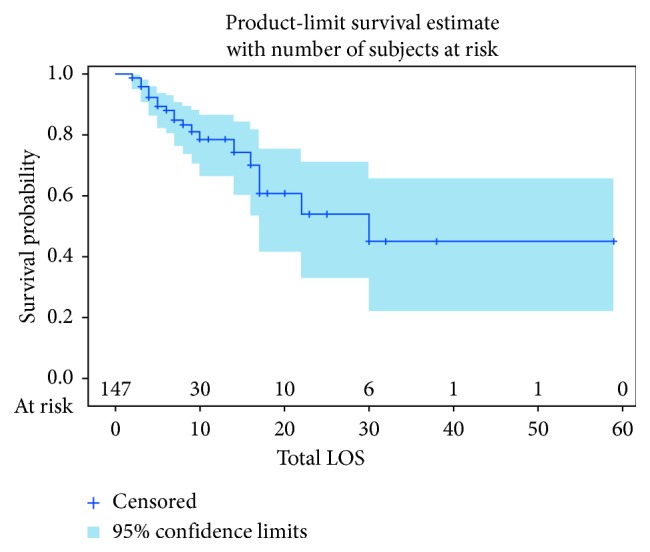
Kaplan–Meier curve of survival probability based on length of stay for both groups combined.

**Figure 2:**
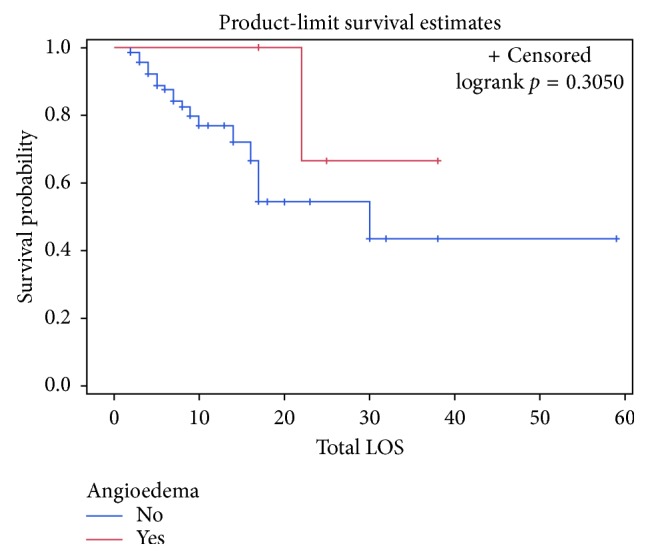
Kaplan–Meier curve of survival probability based on length of stay between the two groups.

Overall, total length of stay ranged from two days to fifty-nine days. The median total length of stay for the severe angioedema group was thirty days compared to twelve in the nonangioedema group. The mean time to death was twenty-one days in the nonangioedema group and twenty-two days in the severe angioedema group. Twenty-five patients died in total, with twelve during their hospital admission and thirteen during their hospice admission. The Kaplan–Meier curve for total length of stay as a measure for survival probability is shown in Figure 1. The Kaplan–Meier curve for survival probability based on total length of stay between the two groups is shown in Figure 2.

## 5. Discussion

As stated previously, severe tPA-induced angioedema cases are thought to be rare and have been reported in 0.18 to one percent of all patients receiving tPA for ischemic CVA indications [1–5, 10, 13, 17]. This study found an incidence of 2.7 percent for severe tPA-induced angioedema and therefore would postulate that the incidence of severe angioedema is more frequent than previously reported. This discrepancy may be related to incomplete data from previous studies as not all studies available on tPA-induced angioedema delineate between mild and severe cases and instead report only an overall incidence [9, 12, 14–16, 18, 19]. Of those that did delineate between severities and found a lower incidence of severe angioedema, this may be related to an overall larger population size in those studies [1, 3–5, 10, 13, 17].

As expected, as length of stay increases with an ischemic CVA, there is a decrease in probability of survival, which is shown by the Kaplan–Meier curve in Figure 1. Additionally, development of severe angioedema was found to have a longer total length of stay. One could reason that a longer length of stay for an ischemic CVA indicates a more serious CVA and/or more serious complications, such as angioedema and need for endotracheal intubation seen in this study. Interestingly, the Kaplan–Meier curve in Figure 2 shows that the severe angioedema group has a higher probability of survival. While it would be unreasonable to infer that development of severe angioedema is actually protective based on such a small population that developed severe angioedema, it suggests that overall the development of severe angioedema does not worsen survival.

As noted, bradykinin B2 receptors are upregulated in dying neurons [1]. To this effect, a longer time between symptom onset and tPA administration could result in an increase in dying neurons which would increase the amount of bradykinin B2 receptors and thus increase the risk of angioedema. This study showed a shorter overall mean time from symptom onset to needle time (140 min.) than the studies by Hurford et al. (156 min.) and Hill and Buchan (155 min.) [1, 10]. The opposite to this proposed theory was found in this study as despite the shorter mean time from symptoms onset to needle time seen in this study, the incidence of severe angioedema was higher than in Hurford et al. and Hill and Buchan [1, 10]. Comparisons between other studies that delineated between mild and severe angioedema are not possible as those studies did not report data on time from symptom onset to tPA administration [2–5, 13, 17]. Further examination of this discrepancy lends to the comparison of NIHSS scores suggesting a more severe CVA would portend an increased risk of developing severe angioedema. This is explained similarly as before with increased bradykinin B2 receptors available on the onset of symptoms in more severe CVAs that are able to interact with tPA, thus increasing the risk of angioedema. The median NIHSS score for patients with severe angioedema in this study was ten. The median NIHSS score for all angioedema patients in Hurford et al. was twelve and in Hill et al. was eighteen. These studies do not differentiate the median NIHSS score for only severe angioedema cases but are likely to be higher than in this study and consequently would not explain this discrepancy. On the other hand, this discrepancy may demonstrate that time from symptom onset to needle time may not be a primary factor on whether or not severe angioedema develops. If nothing else, this discrepancy indicates that more studies are needed to further determine if timing affects the development of severe angioedema.

In regards to tPA-induced angioedema of all severities, previous studies have suggested an increased incidence in those with concomitant use of ACE inhibitors and with MCA territory distribution. For ACE inhibitors, the OR has been reported from 2.3 to as high as 37 [1–3, 17]. An OR of 7.73 was found in this study. Despite a wide confidence interval seen in this study, the findings are consistent with earlier studies proposing an increased likelihood of developing angioedema when combined with tPA for ischemic CVA. For this reason, ACE inhibitors should be stopped, if possible, before administration of tPA, and ACE inhibitors in themselves should be considered as a main risk factor for the development of tPA-induced angioedema.

For MCA distribution, it has been reported that ischemic CVAs involving the insular and frontal cortex can increase the risk of developing angioedema [3, 4, 13, 17]. The study by Hill et al. reports a RR of 4.8 (*p*=0.021) for angioedema in CVAs affecting the insular cortex and 6.4 (*p*=0.010) in CVAs affecting the insular cortex, M1, or M4 territories of the MCA [3]. Unfortunately, distinct involvements of these areas were not documented, and so we are unable to confirm this despite the majority of patients with severe angioedema having MCA involvement.

### 5.1. Strengths

The strengths of this study include a focus on severe tPA-induced angioedema in addition to a thorough review of the literature on the topic of tPA-induced angioedema of all severities and particularly in that of severe cases. Secondly, this study includes an additional important data point of time of symptom onset to tPA administration in relation to development of angioedema which has not been evaluated in the majority of other studies to date [2–9, 13–17, 19]. Additionally, it includes ACE inhibitor use and area of infarct to allow for comparisons to already established notions of tPA-induced angioedema.

### 5.2. Limitations

Limitations of this study include that it is a retrospective study with the limitations of using the electronic medical record. For example, it is difficult to ascertain from chart review whether the degree of angioedema was severe enough to require intubation or whether intubation was performed preemptively in the event airway compromise was to occur. Additionally, as chart reviews are dependent on health-care providers being able to identify and then adequately document their findings, mild cases of angioedema very likely would be missed with this study design. Therefore, evaluation of the overall incidence of tPA-induced angioedema would be inaccurate, establishing the need for more prospective studies on this topic to better evaluate this overall incidence. Secondly, the sample size is small with only four patients developing severe angioedema. With a small population that developed severe angioedema, it is difficult to make comparisons between this group and the patients who did not develop angioedema. Lastly, to be included in this study, the patient had to have received tPA for suspected CVA as opposed to confirmed CVAs used in other studies reviewed [1–4, 6, 7, 9, 10, 13, 17]. As stated previously, cerebral ischemia increases the amount of B2 bradykinin receptors in the brain which can increase the risk of developing angioedema with tPA, but it is unclear if this increase in receptors occurs only in ischemic CVAs or in transient ischemic accidents (TIAs) as well. For that reason, the patients that did not have imaging-confirmed CVAs may be at lower risk of developing severe tPA-induced angioedema which could make the incidence appear lower than it actually is. Additionally, in some of the studies reviewed, CVA mimics (i.e., seizures, complicated migraines, and conversion disorder) were not found to have any episodes of angioedema placing these patients at lower risk of developing severe angioedema and suggesting that some degree of ischemia is necessary to develop severe angioedema [14, 18, 19]. Taking this into account, there were one hundred twelve imaging-confirmed CVAs which would place the incidence of severe tPA-induced angioedema higher at 3.6 percent (95% CI: 1.2% to 8.4%). Again though, it is unclear if TIA upregulates bradykinin B2 receptors and increases the risk of angioedema. This new incidence of 3.6 percent then would not account for patients that may have had a TIA, as by definition a TIA does not show changes on CT or MRI, which would increase the total sample size. Additionally, it is unclear then which patients had TIA or which had CVA mimics as both would have negative imaging and omission of CVA mimics would lead to a smaller population size and increased incidence. With these considerations, it is reasonable to place the true incidence of severe tPA-induced angioedema somewhere between 2.7 and 3.6 percent, still higher than the incidence found in the literature.

## 6. Conclusions

In conclusion, severe angioedema remains a life-threatening adverse effect of tPA in ischemic CVAs and the incidence found in this study suggests a higher incidence than what was previously reported. Interestingly, this study shows that a shorter time between tPA administration and symptom onset increases the risk of developing severe angioedema. Unfortunately, the cause for this was not elucidated and therefore will need to be examined in future studies as the majority of the current studies do not report on this important variable. Finally, while this study supports earlier findings of an increased risk of tPA-induced angioedema with ACE inhibitor use and suggests consideration should be taken on stopping the ACE inhibitor or withholding tPA, it more importantly shows that development of severe angioedema results in increased LOS without compromising overall survival, which has not been shown in previous studies.

## Figures and Tables

**Table 1:** Baseline characteristics of all patients by angioedema status.

	Angioedema, *n* (%), *n*=4	No angioedema, *n* (%), *n*=143
Age (years)	79.0 (68.0–87.0)	68.8 (28.0–98.0)

*Sex*		
Male	0 (0)	64 (45)
Female	4 (100)	79 (55)

*Race*		
Caucasian	3 (75)	93 (65)
Black	1 (25)	27 (19)
Asian	0 (0)	3 (2)
Hispanic	0 (0)	0
Unknown	0 (0)	20 (14)

*Coronary artery disease (CAD)*		
Yes	1 (25)	34 (24)
No	3 (75)	109 (76)

*Diabetes*		
Yes	1 (25)	34 (24)
No	3 (75)	109 (76)

*Atrial fibrillation*		
Yes	2 (50)	44 (31)
No	2 (50)	99 (69)

*Hypertension*		
Yes	4 (100)	125 (87)
No	0 (0)	18 (13)

*ACE inhibitor use*		
Yes	3 (75)	40 (30)
No	1 (25)	103 (70)

Median NIHSS score	10	9

Mean symptom onset to needle time (minutes)	118.3	140.9

*Area of ischemia on imaging*		
Basal ganglia	0 (0)	6 (4)
Middle cerebral artery (MCA) only	3 (75)	77 (54)
Anterior cerebral artery (ACA) only	0 (0)	6 (4)
Posterior cerebral artery (PCA)	1 (25)	17 (12)
Both MCA and ACA	0 (0)	2 (1)
None evidenced	0 (0)	35 (25)

Median total length of stay (days)	30	12

*Death*		
Yes	1 (25)	24 (17)
No	3 (75)	119 (83)

**Table 2:** Number of patients that developed severe angioedema with ACE inhibitor use and other comorbidities.

	Angioedema/total	Odds ratio	CI
ACE inhibitor use	3/43 (7.0%)	7.72	0.59–409.35
CAD	1/35 (2.9%)	1.07	0.01–13.81
DM	0/43 (0.0%)	0.00	0.00–2.69
Atrial fibrillation	2/46 (4.3%)	2.25	0.16–31.77
HTN	4/129 (3.1%)	Infinity	0.12–infinity

## Data Availability

The data used to support the findings of this study are available from the corresponding author upon request.
